# Malignant Müllerian Adenocarcinoma Manifesting With Cardiac Tamponade and Pleural Effusion

**DOI:** 10.7759/cureus.16233

**Published:** 2021-07-07

**Authors:** Naji Maaliki, Spencer Streit, Amy Roemer, Peter Staiano, Anwer Siddiqi, Hadi Hatoum

**Affiliations:** 1 Internal Medicine, University of Florida College of Medicine – Jacksonville, Jacksonville, USA; 2 Internal Medicine, University of Florida College of Medicine – Jacksonville, Jacksonville , USA; 3 Pulmonary and Critical Care Medicine, University of Florida College of Medicine – Jacksonville, Jacksonville, USA; 4 Pathology, University of Florida College of Medicine – Jacksonville, Jacksonville, USA; 5 Pulmonary and Critical Care Medicine, University of Florida Health – Jacksonville, Jacksonville, USA

**Keywords:** cardiac tamponade, pericardial effusion, pleural effusion, malignant effusion, endometrial cancer

## Abstract

A 54-year-old woman with a past medical history of untreated stage IV Müllerian adenocarcinoma presented for dyspnea. She was found to have a large right-sided pleural effusion through basic radiology and clinically improved after a CT-guided therapeutic thoracocentesis. However, the patient rapidly deteriorated shortly afterward. A broader workup that included echocardiography revealed a large pericardial effusion with tamponade physiology. The patient underwent an emergent pericardiocentesis, which briefly improved hemodynamics, but her clinical status kept declining until she eventually expired. Subsequent cytology of the pleural and pericardial fluid revealed malignant cells of Müllerian origin.

## Introduction

Cardiac tamponade is an emergency that occurs when an excessive fluid accumulation within the pericardial cavity compresses the heart and impairs its function [1]. Metastatic pericardial tamponade is a rare complication of Müllerian cancers that can prove fatal if it goes unrecognized [1]. Müllerian malignancies are neoplasms that arise from cells of Müllerian origin, which denotes the embryonic basis of the female reproductive tract, developing into the fallopian tubes, uterus, uterine cervix, and superior aspect of the vagina [2]. These carcinomas may be classified as epithelial ovarian, tubal, and peritoneal cancers [3]. Common sites of metastasis include the pelvic and para-aortic lymph nodes, vagina, peritoneum, and lungs [4]. Very rarely are cancers of Müllerian origin found in the pericardium [5]. Described here is an unsuspecting case of cardiac tamponade physiology in a critically ill woman with metastatic Müllerian adenocarcinoma requiring emergent pericardiocentesis. Unexplained dyspnea in patients with malignancy requires a high index of suspicion to rule out a pericardial effusion. 

## Case presentation

**Figure 1:**
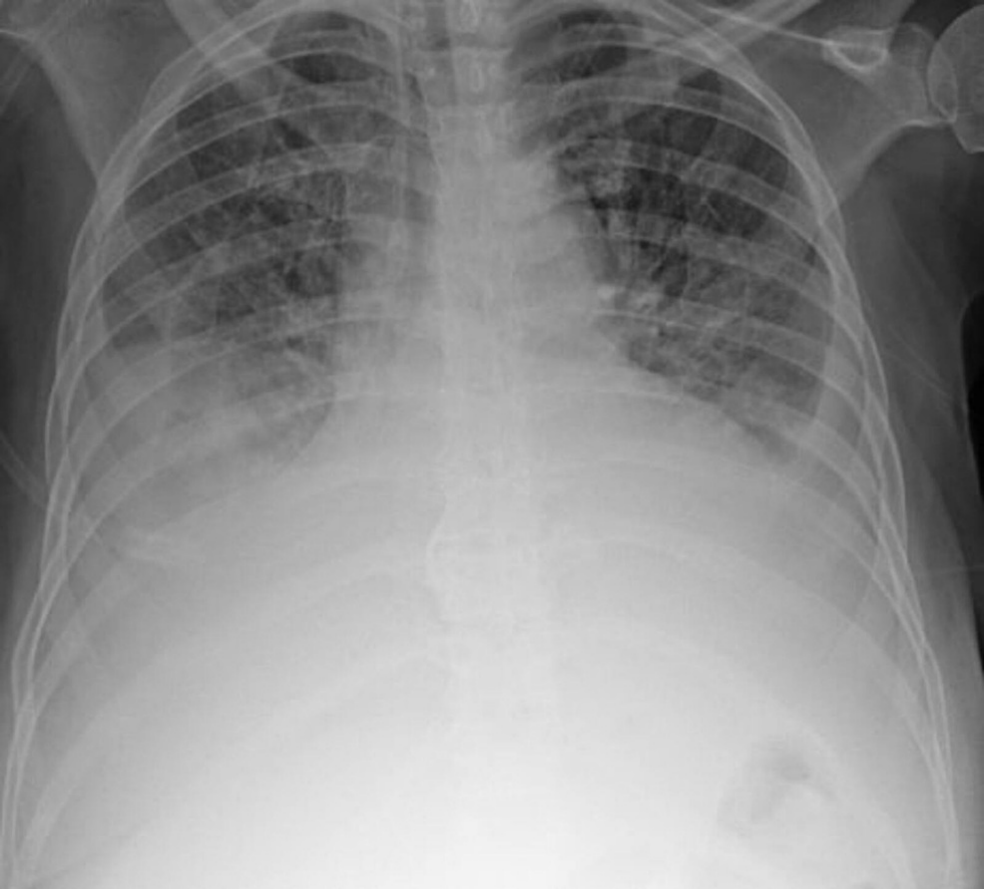
Initial chest X-ray showing bilateral pleural effusions with a significant right pleural effusion, cardiomegaly, and bilateral opacities.

**Figure 2:**
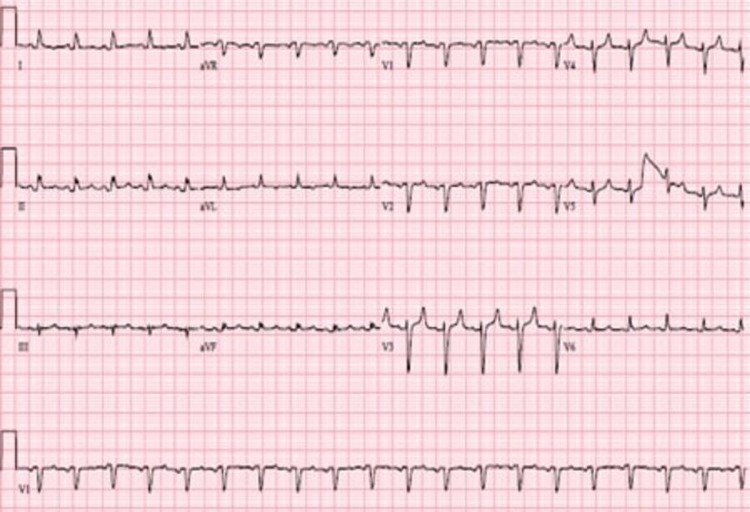
ECG demonstrating low-voltage QRS and sinus tachycardia.

**Figure 3:**
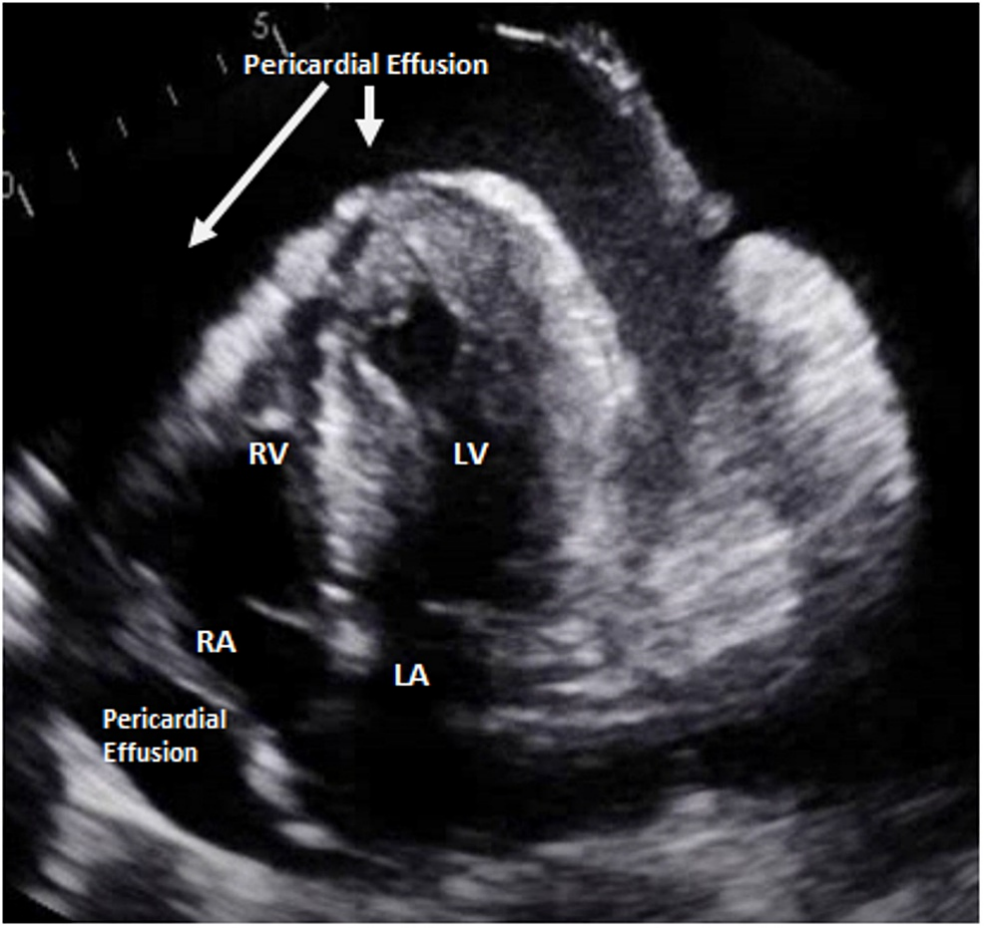
Four-chamber echocardiography demonstrating large pericardial effusion and right chamber collapse. RV, right ventricle; LV, left ventricle; RA, right atrium; LA, left atrium.

**Figure 4:**
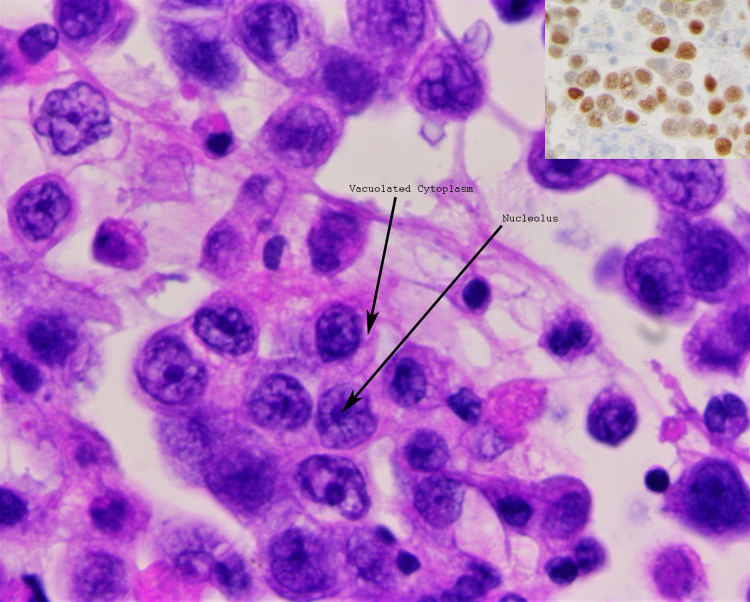
Hematoxylin and eosin stain of the pericardial fluid demonstrating adenocarcinoma cells with high nuclear-to-cytoplasmic ratio, prominent nucleoli, and delicate vacuolated cytoplasm. Inset (top right) demonstrates positive PAX-8 staining for cells of Müllerian origin.

A 54-year-old woman with a recent diagnosis of stage IV Müllerian adenocarcinoma and end-stage renal failure requiring hemodialysis presented to our institution with dyspnea. She was recently diagnosed with her cancer three weeks before presentation after presenting for fatigue, weight loss, and lower abdominal pain for the past few months. A Papanicolaou test was suggestive of adenocarcinoma, and a follow-up CT of the abdomen and pelvis showed innumerable enlarged lymph nodes, including the paratracheal, supraclavicular, axillary, external and internal iliac lymph nodes, alongside a moderate right pleural effusion, a small left pleural effusion, and a small-sized pericardial effusion. A subsequent diagnostic thoracentesis with cytology confirmed metastatic adenocarcinoma of Müllerian origin. The commencement of her treatment was still pending insurance attainment. On admission, she complained of progressive shortness of breath for a week associated with right-sided chest-wall pain, exacerbated by inspiration. Physical examination revealed tachypnea, tachycardia, decreased breath sounds bilaterally with dullness to percussion over the right lower lung zone, and bilateral lower limb edema. Workup was significant for an elevated white blood cell count of 13 x 10^9 ^cells/L and a creatinine of 2.32 mg/dL. Chest X-ray displayed bilateral pleural effusions with the right markedly larger than the left, bilaterally increased bronchovascular markings, and cardiomegaly (Figure 1). Initial treatment included broad-spectrum antibiotics and a CT-guided diagnostic and therapeutic thoracocentesis with the removal of 1 L of serous pleural fluid, leading to a brief improvement in symptoms. However, two days later, she acutely deteriorated with the development of hypotension, tachycardia, encephalopathy, and respiratory failure. New physical examination findings revealed jugular venous distention, decreased heart sounds, and decreased peripheral pulses. A repeat chest X-ray was essentially unchanged, with a very slightly decreased right-sided pleural effusion, similar-sized cardiac silhouette, and no visible pneumothorax. An ECG revealed sinus tachycardia and low-voltage QRS (Figure 2). Echocardiography demonstrated a large pericardial effusion measuring 3 cm along the apical border, with invagination of the right ventricular free wall during late diastole and an excessive respiratory variation of the tricuspid and mitral inflow velocities suggestive of cardiac tamponade (Figure 3, Video 1). She was subsequently intubated and fluid resuscitated, and required norepinephrine for persistent hypotension. Urgent pericardiocentesis was performed, and 533 mL of bloody pericardial fluid was removed. The patient’s vital signs briefly improved, but then her status continued to decline despite increasing ventilatory and vasopressor support, and she eventually expired. Cytology of both the pleural and pericardial fluid specimens revealed metastatic poorly differentiated adenocarcinoma, favoring a Müllerian primary based on immunohistochemistry (Figure 4). 

**Video 1:** Four-chamber echocardiography demonstrating large pericardial effusion and right chamber collapse.

## Discussion

Cardiac tamponade is the detrimental compression of the heart by an accumulation of fluid, blood, gas, or pus in the pericardial space. Etiologies are commonly infectious, neoplastic, traumatic, idiopathic, or any progression of pericarditis. Incidence rates are challenging to attain due to high mortality [1]. Tamponade occurs when the pericardial content, normally a serous fluid of less than 30 mL, increases to a point where the pericardial pressures exceed the intracardiac pressures, leading to impaired systemic venous filling of the right ventricle and a subsequent decrease in cardiac output [6]. Further deterioration in cardiac output occurs as the intrapericardial pressure approaches the left ventricular pressure [6]. Clinically, patients present with hypotension, tachycardia, respiratory distress, alongside an elevated jugular venous pressure, muffled heart sounds, and cold extremities [7]. A specific finding includes pulsus parodoxus, where the systolic blood pressure decreases more than 10 mmHg with inspiration [1,7]. ECG can display a low-voltage QRS complex, electric alterans, and acute pericarditis findings when pericarditis is suspected to be the most likely etiology. Tamponade remains a clinical diagnosis, but can be supported by echocardiographic findings of a dilated inferior vena cava, early right ventricular diastolic collapse, and late right atrial diastolic collapse [1,6]. As the pericardial pressure begins to equilibrate with all intracardiac pressures, this leads to abnormal ventricular septal motion, elevated respiratory variability in mitral inflow velocity, respiratory variation in ventricular chamber size, and aortic outflow velocity [6,7]. Treatment is an urgent pericardiocentesis to relieve the cardiac compression and obstructive shock, alongside aggressive fluid resuscitation and potentially vasopressors for hemodynamic support [1-7].

Malignancy can occasionally present with both pericardial and pleural effusions. According to a recent study [8], the most common cause of these effusions is a thoracic neoplasm such as a lung or breast tumor, with lung adenocarcinoma as the most common cause. Very rarely, endometrial cancer has been associated with pericardial effusions. Only five such cases have been reported in the literature review through 2019 [5]. The pathophysiology is thought to be from the lymphatic spread of malignant cells from the Müllerian source to the mediastinal lymph nodes, which the pericardium drains into from the subepicardial plexus, leading to retrograde tumor cell migration [9]. The rapid production of vascular growth factors from the neoplastic cells can potentially increase vascular permeability and promote leakage, thus producing an effusion [10]. The severity of pericardial effusion depends on the rate of fluid accumulation, and, therefore, chronic malignant effusions may present with nonspecific symptoms of dyspnea and chest discomfort [11]. In addition, an effusion leading to tamponade physiology should be considered. Echocardiography, therefore, is an essential tool in the assessment of patients with malignancy presenting with dyspnea, as it can detect tamponade early on, which would necessitate lifesaving pericardiocentesis. While pericardiocentesis may not be necessarily needed in asymptomatic effusions, it should be considered in large effusions of unknown etiology or in patients with an underlying disease known to cause pericardial effusions, as this can provide both diagnostic and therapeutic benefits [12,13]. The diagnostic gold standard of malignant effusions is through cytopathology, which can identify neoplastic cells, aided by additional findings of elevated proteins, negative microbial cultures, and increased inflammatory cells [13]. Malignant pericardial effusions are commonly associated with an unfavorable prognosis and usually recur, often necessitating a pericardial window [13].

In our case, the patient experienced both a pleural and pericardial effusion, the latter being the probable cause of death from tamponade. Incidence of such malignant effusions is uncommon, let alone both occurring together. This case highlights the potential sequelae of an unmanaged malignancy, the need for a broad differential diagnosis in such situations, and a high index of suspicion for lethal complications, including cardiac tamponade.

## Conclusions

Cardiac tamponade is a lethal occurrence that must be rapidly diagnosed and treated with pericardiocentesis to prevent hemodynamic collapse. This case is unusual given the rare occurrence of pericardial tamponade as a late complication of gynecological malignancy. A high index of suspicion for pericardial tamponade must be maintained in these patients presenting with respiratory complaints. 
